# Infantile idiopathic intracranial hypertension: case report and review of the literature

**DOI:** 10.1186/s13052-021-01191-5

**Published:** 2022-01-10

**Authors:** Francesco Del Monte, Laura Bucchino, Antonia Versace, Irene Tardivo, Emanuele Castagno, Giovanni Pieri, Giulia Pilloni, Enrico Felici, Antonio Francesco Urbino

**Affiliations:** 1 Department of Public Health and Pediatrics, University of Turin, Regina Margherita Children’s Hospital, A.O.U. Città della Salute e della Scienza di Torino, Piazza Polonia 94, 10126 Turin, Italy; 2 Division of Pediatrics, Department of Health Science, University of Piemonte Orientale, Novara, Italy; 3 Department of Pediatric Emergency, Regina Margherita Children’s Hospital, A.O.U. Città della Salute e della Scienza di Torino, Turin, Italy; 4 Pediatrics and Pediatric Emergency Unit, The Children’s Hospital, AO SS Antonio e Biagio e C. Arrigo, Alessandria, Italy; 5 Division of Pediatric Neurosurgery, Regina Margherita Children’s Hospital, A.O.U. Città della Salute e della Scienza di Torino, Turin, Italy

**Keywords:** Idiopathic intracranial hypertension, Pseudotumor cerebri syndrome, Infant, Papilloedema, CSF opening pressure

## Abstract

**Background:**

Idiopathic intracranial hypertension is an infrequent condition of childhood, and is extremely rare in infants, with only 26 cases described. The etiology is still unknown. Typical clinical manifestations change with age, and symptoms are atypical in infants, thus the diagnosis could be late. This is based on increased opening pressure at lumbar puncture, papilloedema and normal cerebral MRI. The measurement of cerebrospinal fluid opening pressure in infants is an issue because many factors may affect it, and data about normal values are scanty. The mainstay of treatment is acetazolamide, which allows to relieve symptoms and to avoid permanent visual loss if promptly administered.

**Case presentation:**

We report the case of an 8-month-old infant admitted because of vomit, loss of appetite and irritability; later, also bulging anterior fontanel was observed. Cerebral MRI and cerebrospinal fluid analysis resulted negative and after two lumbar punctures he experienced initial symptom relief. Once the diagnosis of idiopathic intracranial hypertension was made, he received oral acetazolamide, and corticosteroids, with progressive symptom resolution.

**Conclusions:**

Infantile idiopathic intracranial hypertension is extremely rare, and not well described yet. Bulging anterior fontanel in otherwise healthy infants with normal neuroimaging should be always considered suggestive, but can be a late sign, while irritability and anorexia, especially if associated with vomiting, may represent an early sign. In such cases, lumbar puncture should be always done, hopefully with cerebrospinal fluid opening pressure measurement, which is among coded diagnostic criteria, but whose threshold is controversial in infants. Early diagnosis, timely treatment and strict follow-up help to prevent vision loss or death of affected infants.

## Background

Idiopathic intracranial hypertension (IIH) is a clinical syndrome caused by elevated cerebrospinal fluid (CSF) pressures with no evidence of mass lesion on neuroimaging. It is infrequent in childhood, and extremely rare in infancy [1–5]. We describe the case of an 8-month-old male affected by IIH, followed by a brief review of the literature.

## Case presentation

A previously healthy 8-month-old infant with uneventful personal history and regular psychomotor development was admitted to the Emergency Department because of non biliary vomiting, irritability and lethargy; 4 days before, he had fallen from the bed without any apparent acute complications; no visit was done in that occasion.

On the physical examination at the arrival, he was fussy, with inconsolable crying, but no neurological sign was observed. Anterior fontanel was normal. Laboratory tests included blood cell count, erythrocyte sedimentation rate, C-reactive protein, liver and renal function tests, and urine analysis, all proving negative. Intussusception was excluded on abdominal ultrasound. Head ultrasound showed mild frontal subarachnoidal enlargement, up to 5 mm.

Soon after the admission, he got worse, with bulging anterior fontanel and anorexia. Head computed tomography (CT) scan proved negative for intracranial lesions, and the electroencephalogram was normal. Then the infant underwent lumbar puncture (LP) on sedation. LP was performed in the lateral recumbent  position, with legs flexed. After the infant’s back was cleaned with antiseptic solution, 22-gauge spinal needle was inserted at the 4th lumbar space. In this case we had not the possibility to measure CSF opening pressure, anyway we used an empiric method already described in literature [6, 7], and we observed that in 10 s the number of CSF drops flowing through the needle was higher compared to the number observed during routine procedure. After the needle was removed, the area was cleaned and a compressive medication on the needle site was performed. The infant was put on i.v. ceftriaxone and acyclovir after the CSF sample collection, then CSF analysis revealed slightly increased protein level (74,6 mg/dl, normal value 20–45 mg/dl) and leucocyte level (6 cells/mmc), with normal glucose level and negative Gram stains and cultures, as well as negative HSV, EBV, CMV, Enteroviruses, and SARS-CoV-2 polymerase chain reaction. Immediately after LP, the infant’s condition improved significantly, and he could be fed properly.

The ophthalmology evaluation revealed pale and elevated optic disc with retinal venous congestion.

Brain magnetic resonance imaging (MRI) with gadolinium showed mild narrowing of the pituitary gland peduncle and flattening of the *sella turcica*. The CSF flow study was normal and venography study did not show any venous thrombosis. Under suspicion of IIH, complete blood metabolic, endocrinological, and infective workout proved negative.

Five days after the LP, the patient presented again irritability and anorexia; head circumference increased from 46.5 cm to 47.7 cm. The patient was then transferred to the regional Pediatric Hospital for neurosurgery evaluation and further investigation and therapy. His neurologic status and physical examination were normal except for full, tense and pulsing anterior fontanel measuring 3 × 3 cm. A new ophthalmology evaluation confirmed papilloedema, and another LP was done using the same method: again, opening lumbar pressure was increased, and CSF analysis was normal. West Nile Virus polymerase chain reaction was also negative. Neurosurgery evaluation did not suggest any other intervention.

IIH was then diagnosed on Friedman criteria (Table 1) [8] and the infant was put on acetazolamide (10 mg/kg/day) and i.v. dexamethasone (0.2 mg/kg/day). After the second LP and the beginning of medical therapy, he rapidly got better, anterior fontanel flattened, and vital signs remained normal during all the admission.

**Table 1:** Diagnostic criteria for idiopathic intracranial hypertension syndrome (IIH) (adapted from Friedman DI, et al., 2013) [8]

**Diagnostic criteria for IIH: A-E required** **a.** Papilloedema **b.** Normal neurologic examination, except for cranial nerve abnormalities **c.** Neuroimaging: normal brain parenchyma without evidence of hydrocephalus, mass, or structural lesion and no abnormal meningeal enhancement on MRI, with and without gadolinium, for typical patients (female and obese), and MRI, with and without gadolinium, and magnetic resonance venography for others; contrast-enhanced CT may be used if MRI is contraindicated or unavailable* **d.** Normal CSF composition **e.** Elevated LP opening pressure (> 25 cmH2O in adults; > 28 cmH2O in children; > 25 cmH2O CSF if the child is not sedated and not obese) **Diagnostic Criteria for IIH without Papilledema** **a.** B-E from above PLUS unilateral or bilateral VI nerve palsy **b.** Diagnosis may be suggested, but not made in absence of VI nerve palsy nor papilloedema if B-E criteria from above satisfied AND at least 3 of the following neuroimaging criteria are satisfied: 1. Empty sella 2. Flattening of the posterior aspect of the globe 3. Distention of the perioptic subarachnoid space 4. Transverse venous sinus stenosis	
**A diagnosis of IIH syndrome is definite if the patient fulfills criteria A-E.** **The diagnosis is considered probable if criteria A-D are met, but the measured CSF pressure is lower than specified for a definite diagnosis.** * Signs of elevated intracranial pressure frequently found on MRI: perioptic subarachnoid space distension and empty sella.	

Further investigations revealed normal calcium metabolism, decrease level of vitamin D 25-OH (21 ng/ml, normal value > 30 ng/ml), and increased Vitamin A level (1.03 μg/ml, normal value 0.14–0.52 μg/ml).

Dexamethasone was stopped after 10 days, and acetazolamide was gradually decreased in 2 months without any adverse effects, and full recovery.

## Discussion and conclusions

Pseudotumor cerebri syndrome (PTCS) is a rare condition described for the first time in 1893 [9], with elevated intracranial pressure, normal CSF analysis, and without intracranial lesions on neuroimaging. PTCS can be primary (also known as IIH), if no cause is identified, or secondary, if a specific cause is recognized (i.e. drugs administration, abnormalities of the cerebral venous system, or predisposing systemic diseases).

Though PTCS can affect both children and adults, there are significant differences regarding the clinical spectrum, risk factors and epidemiology. In children, the incidence is 0.6–0.9/100,000/year [10], and it is very rare in infancy. To our knowledge, only 26 infants have been described so far; among them only 6 met all the diagnostic criteria [1–5]. In order to find literature on already described cases of infantile PTCS, we searched Pubmed from inception to February 2021; age limit was 0–23 months and we used the following terms combined with boolean operators AND and OR: *idiopathic intracranial hypertension*, *infantile idiopathic intracranial hypertension*, *pseudotumor cerebri*, *infants*. Out of 354 articles, 143 were case reports, reviews and systematic reviews; finally, 17 papers describing 26 infants were included in our brief review.

IIH is the most common kind of PTCS both in children and adults [11]. Though female gender, obesity, and polycystic ovary represent risk factors for IIH in pubertal children, this is not shown in younger ones, particularly in infants [12]. Among the 26 infants with IIH described in literature, a couple of twins were affected by cystic fibrosis and Vitamin A deficiency, one had isolated Vitamin A deficiency, four had Vitamin D deficiency, two had positive serum human herpesvirus-6 testing, two had hypophosphatasia, and four had a history of topical hydrocortisone treatment some days before the onset of symptoms. In our case, elevated serum Vitamin A level was found.

The clinical manifestations of IIH vary with age. Headache is a key symptom, described as progressive frontal pain worsening with Valsalva maneuver and postural changes, but it is reported only by 36.6% of pediatric patients [13]. Other symptoms include nausea, vomiting, neck pain, tinnitus, visual impairment and diplopia secondary to VI cranial nerve palsy [14].

Younger children may show nonspecific symptoms like irritability, hyporeactivity, anorexia, sleep disruption, head tilting, and less often papilledema. Infants may present a bulging fontanel and irritability as the most commonly reported initial symptoms, as well as vomiting [1–5]. Another unequivocal sign of intracranial hypertension is papilloedema, however it was reported only in 9 of the 26 infants described in literature.

Diagnostic criteria were firstly formulated by Dandy in 1937 [15], then Smith re-proposed them in 1985 [16], as follows: 1) signs and symptoms of intracranial hypertension, 2) localized neurological signs (except paralysis of the VI pair of cranial nerves), 3) CSF opening pressure > 25 cmH_2_0, with normal examination, 4) negative head CT scan.

A new revision was published in 2013 (Table 1): the new criteria are more restrictive and differentiate the diagnosis into confirmed IIH (papilloedema and increased CSF pressure), probable IIH (papilloedema and normal CSF pressure), and IIH without papilloedema (only increased pressure with VI cranial nerve palsy) [8]. Therefore, ophthalmological evaluation of *fundus oculi*, LP and neuroimaging are required to make a diagnosis. The ophthalmological evaluation aims to identify papilloedema and should be performed by a pediatric specialist. Typically, the optic disc is pale and oedematous, with surrounding tortuous and dilated retinal vessels [17].

MRI of the brain is necessary to exclude any lesion that could produce intracranial hypertension, and should include contrast medium infusion and angiographic study [8]. Moreover, characteristic radiological signs are: *sella turcica* with narrowed pituitary peduncle, as observed in our infant, and crushing of the posterior pole of the eye [8].

The LP, which is both a diagnostic and a therapeutic procedure itself, must be performed whenever intracranial hypertension is suspected, after excluding possible contraindications. Not only the opening pressure, but also CSF analysis is important for diagnostic purposes. In our infant, no abnormal CSF values were found, except for proteins slightly higher than normal.

Even if the measurement of CSF opening pressure could be an important additional diagnostic factor, actually it is not routinely performed [18]. In our case, the neurosurgeon described the opening pressure as increased on both occasions by counting the number of CSF drops flowing through the spinal needle in a specified period, as already described [6, 7], however we do not know the precise pressure measurement. In published studies on PTCS, CSF opening pressure was not documented for all the patients, because other factors documented indirectly elevated CSF pressure (symptoms, imaging data) [8, 19]. Our infant presented with papilloedema and bulging anterior fontanel, which represent indirect signs of high CSF pressure. Indeed, data about CSF opening pressure in childhood are scanty and come mainly from patients ageing 1–18 years [20, 21], but to our knowledge no clear threshold has been established in infants < 1 year, in which the open fontanel and sutures might influence the measure. Moreover, other factors may affect the opening pressure value: in particular sedation-related hypercapnia may raise it, as well as crying in not sedated children [8, 20]. For these reasons, a cut-off of 28 cmH_2_O could be useful more for research than for clinical purpose [20]. In his article, Ellis concluded that the normal range for CSF opening pressure measured in a flexed lateral decubitus position in children is 10 to 28 cm H_2_O [6]. However, Avery et al. presented a case to describe the inaccuracy of CSF opening pressure measures by LP: they concluded that a single CSF opening pressure value should not be considered as the only determinant of elevated intracranial pressure [20]. On the other hand, low opening pressure should not exclude the diagnosis of PTCS if typical symptoms and papilloedema are reported [8].

Principles of therapy in children are based on guidelines for adult, aiming mainly to prevent permanent loss of vision and relieve from symptoms (above all irritability in infants, and headache in older children). The therapeutic cornerstone is acetazolamide, a carbonic anhydrase inhibitor that reduces the production of CSF; in a recent study, acetazolamide was effective in 76% of children with IIH [21]. The recommended starting dose is 10–20 mg/kg/day, which can be progressively increased up to 100 mg/kg or 2 g per day. Side effects are mild metabolic acidosis, fatigue, dysgeusia and paraesthesia. In case of failure or contraindications to give acetazolamide, topiramate can be used, thanks to its weak inhibitory action on carbonic anhydrase [22].

I.v. corticosteroids could be prescribed in association with acetazolamide, in case of severe visual impairment at the onset. However, currently acetazolamide alone is preferred also in this case, due to fewer side effects [23]. In our case, the corticosteroid was administered for anti-oedema purposes in association with acetazolamide, in consideration of poor general conditions and marked irritability.

The optimal duration of therapy is not well defined, but it should be continued until complete resolution of the papilloedema. A recent review of pediatric cases shows that the course of the disease is highly variable, usually showing remission after more than 7 months [1]. In our case, the infant was treated for 2 months with acetazolamide, with complete resolution of papilloedema and of symptoms 3 months after discharge.

In case of rapid worsening of visual function despite adequate medical therapy, neurosurgical interventions may be needed, such as fenestration of the optic nerve, or lumboperitoneal or ventriculoperitoneal shunt [24].

The main complication of untreated IIH is permanent vision loss (up to 20% of children), so prompt therapy and strict pediatric and ophthalmological follow-up is required [25].

Negative prognostic factors are visual impairment and severe papilloedema already at the onset. Finally, the risk of recurrence is estimated at 18–20% and is more frequent in the first 18 months after diagnosis [26].

In conclusion, infantile IIH is an extremely rare disease, not yet well described in literature. Of course, affected infants cannot describe typical symptoms such as visual impairment, diplopia, tinnitus, headache and nausea, and this makes the diagnosis difficult and often late. Bulging anterior fontanel in otherwise healthy infants with normal neuroimaging should be always considered suggestive of IIH, but can be a late sign. In accordance with literature, our case shows how irritability and anorexia, especially if associated with vomiting [1], may represent an early sign of intracranial hypertension in infancy. In such cases, LP should be always done, hopefully with CSF opening pressure measurement, which is among coded diagnostic criteria, but whose threshold is controversial in infants. Early diagnosis, timely start of acetazolamide and strict pediatric and ophthalmological follow-up help in reducing the risk of relapse and safeguard the patient’s visual acuity.

## Data Availability

Not applicable.

## Abbreviations

IIH: Idiopathic intracranial hypertension
CSF: Cerebrospinal fluid
LP: Lumbar puncture
CT: Computed tomography
MRI: Magnetic resonance imaging
PTCS: Pseudotumor cerebri syndrome
